# Identifying key challenges and needs in digital mental health moderation practices supporting users exhibiting risk behaviours to develop responsible AI tools: the case study of Kooth

**DOI:** 10.1007/s43545-022-00532-3

**Published:** 2022-09-29

**Authors:** Elena Nichele, Anita Lavorgna, Stuart E. Middleton

**Affiliations:** 1 University of Notthingham, Notthingham, UK; 2 University of Southampton, Southampton, UK; 3 Present Address: University of Bologna, Bologna, Italy

**Keywords:** Digital moderation, Digital counselling, Risk behaviours, Responsible AI, Mental health and wellbeing

## Abstract

Digital platforms for mental health and wellbeing purposes have become increasingly common to help users exhibiting risk behaviours (e.g. self-harming, eating-related disorders) across all ages, opening new frontiers in supporting vulnerable users. This study stems from a larger project, which explores how responsible AI solutions can up-scale existing manual moderation approaches and better target interventions for young people who ask for help or engage in risk behaviours online. This research aims to better understand the challenges and needs of moderators and digital counsellors, i.e. the ‘behind the scenes’. Through this case study, the authors intend to contribute to the development of responsible AI tools that are fit for purpose and better understand the challenges. The key focus lies on Kooth.com, the UK’s leading free online confidential service offering counselling and emotional wellbeing support to young people in the UK through its online web-based and pseudo-anonymous digital platform.

## Introduction

Using social media mechanisms and other digital tools to scale up services and maximise affordances such as anonymity for mental health and wellbeing purposes is not new (e.g., McCosker 2018). On the one hand, over the last 15 years digital platforms have offered an important mean for improving the reach and scale of mental health support, opening new frontiers (Kivitz 2013; National Institute of Mental Health 2017; McCosker 2018). Amongst the several benefits associated with digital technologies in health settings are those associated with information access, empowerment, and the opportunity to find supportive relationships (McCosker and Darcy 2013; Moorhead et al. 2013; Tucker and Goodings 2017; Saha 2020), as well as the possibility to engage with those people hard to reach and support (Tanis 2008; Sokol and Fisher 2016), providing additional help to individuals with urgent or special needs. More generally, the number of people seeking health-related advice (including mental health and wellbeing advice) on digital platforms is increasing, particularly because users appreciate this different style of communication, often leading to emotional care and empathy (Lederman et al. 2014). On the other hand, it has been recognised that the success of digital platforms in health settings depends on a range of support and sociocultural factors (Hansen and Aranda 2012). These approaches alter both the ‘expert-client relationship’ (i.e. how the worker and the client interact ‘around the information sought and given’, as the level of self-disclosure increases online—see Mowlabocus et al. 2015: 5) and the ‘public-professional relationship’ (Kivitz 2013), in the sense that patients and the general public are now able to remain permanently connected with health professionals and institutions (Kivitz 2013), in a way that ‘challenges notions of expertise, whether health, biomedical or cultural, inspiring attempts to mobilise new forms of community-oriented and personalised public health intervention through digitally mediated peer practices” (McCosker 2018, p. 4751).

In a context where mental health organisations have limited funding and hence need to carefully choose how to allocate that funding to support services, it is important to understand how to best design digital tools and to assess their effectiveness (as discussed in McCosker 2018), but also to consider the challenges and needs experienced ‘behind the scenes’ by those relying on these digital tools for their work: we believe this is a necessary step to improve the systems already available.

Our study addresses this latter point by interviewing key actors (involved in moderation, counselling, emotional wellbeing support, or managerial roles), within the frame of a broader research project (the UKRI TAS Hub-funded project *SafeSpacesNLP: Behaviour classification NLP in a socio-technical AI setting for online harmful behaviours for children and young people*, 2021–2022^1^) that will use these insights to explore how ‘responsible AI’ solutions (Ghallab 2019) can support up-scaling existing manual approaches and better target interventions for young people who ask for help or engage in risk behaviours online, which can have a detrimental impact on their physical (e.g. suicide, self-harming, eating-related disorders), mental (e.g. anxiety, depression, sleep disruptions, body image distortions, cyberbullying, and ‘fear of missing out’) and/or sexual health (e.g. forced marriages, sexual exploitation).

As the next section of this paper discusses in more detail, such users can be denominated ‘vulnerable publics’ (McCosker and Wilken 2017), as they experience and share socially sensitive and emotionally charged challenges for which they seek help, through online moderation and digital counselling. Given their role, the professionals involved in online moderation and digital counselling act as frontline service workers, as they provide a blend of care support, health services and ‘feeling management’ (see Hochschild 2003), which can be labelled as affective, emotional and immaterial labour (see McCosker and Darcy 2013; McCosker 2018). Moreover, since the difficulties users are struggling to cope with are often stigmatised, professionals additionally facilitate information flow, fighting social and health marginalisation or exclusion (see Long et al. 2013). Accordingly, moderators and councillors play a key role in aiding (peer and professional-user) cooperation as well as preventing abusive or dangerous behaviours (see Grimmelmann 2015) perpetrated or suffered by the victims they support.

For the purposes of this paper, we consider ‘responsible AI’ as any AI system which follows the UKRI framework for responsible innovation. This includes the key principles of Anticipate, Reflect, Engage, Act (AREA) and will often involve some sort of stakeholder engagement or co-design to consider responsibly the environment and context in which the AI system will be deployed (UKRI 2022). We focus on Kooth.com,^2^ the UK’s leading free online confidential service (active all year, in the afternoon and evening), which offers counselling and emotional wellbeing support to young people in the UK. Through its digital platform, users can browse through self-help materials, seek support or advice on a range of sensitive topics (from bullying to dealing with suicidal thoughts), share their experience through moderated forums, track their thoughts and feelings through personal journals, and access synchronous and asynchronous text-based chats and drop-in sessions with counsellors or emotional wellbeing practitioners.

After a brief critical overview of the literature that has looked at moderation and digital counselling supporting users exhibiting risk behaviours, and a section on our data collection and analytical approach, this article offers a descriptive account of the working practices at Kooth Plc (the wider organisation), focusing both on ‘what works’ and on the main challenges encountered. Departing from these findings, in the conclusions we discuss the possibilities that a responsible AI can offer to overcome these challenges, without losing track of the positives in place. We finally signpost where some of the latest trends in responsible AI today might offer pathways for researchers and practitioners to overcome these challenges.

## Moderation and digital counselling to support users exhibiting risk behaviours

As mentioned before, research on the use of digital platforms for mental health and wellbeing purposes is not new, as both practitioners, researchers, and even policy makers have recognised the potential of these digital tools to support users exhibiting risk behaviours (e.g,. self-harming and eating-related disorders), across all ages (Moessner and Bauer 2012; de la Harpe et al. 2019; Zhou et al. 2021). For instance, with specific reference to young people—as those targeted by the services at the centre of our analysis—, recognising that cyberspace has become a space in which we express ourselves, shape our self-identity, build meaningful relationships and learn (and hence is a space intrinsically linked to our mental health), the Royal Society for Public Health (2017) called for action to promote the positive aspects of social media for young people. This includes access to other people’s health experiences and expert health information, emotional support and community building, self-expression and self-identity, whilst mitigating the potential negatives (such as anxiety and depression, sleep, body image, cyberbullying, and ‘fear of missing out’).

Users who typically look for support on these digital platforms can be considered as ‘vulnerable publics’, as they cohere around socially sensitive and affective issues or experiences, which are often stigmatised (see McCosker and Wilken 2017). In this context, the role of moderators in online communities and digital counsellors (frontline service workers) is of the utmost importance, as they operate in the blurred lines between caring, or health service work, and ‘feeling management’ (Hochschild 2003), in a peculiar type of affective, emotional and immaterial labour (e.g. McCosker and Darcy 2013; McCosker 2018). Overall, these actors act as brokers facilitating information flow, avoiding marginalisation, social and health exclusion, and stigma (Long et al. 2013), as they sustain online communities, help framing and reframing difficult lived experiences, and create and maintain a bridge between the user-base and professionals (McCosker 2018). As such, they have a very complex role, as they need to maintain authority and be perceived as authentic, whilst creating and maintaining trust (McCosker 2018).

Moderation can be defined as the ‘governance mechanisms that structure participation in a community to facilitate cooperation and prevent abuse’ (Grimmelmann 2015: 6). Additionally, how a group is moderated can influence members’ participation, including their creation and maintenance of commitment to the community (Ley 2007). Moderation can take different forms (West 2018; Seering 2020). For the scope of this study, the difference between automatic or manual moderation matters. Over the years, automated ways to moderate social media (ranging from classification and filtering approaches, used for instance to identify hate speech, to more complex digital tools supporting moderation by considering the context of longer conversations, see e.g. Kurrek et al. 2020; Price et al. 2020; Röttger et al. 2021) have been developed by social media companies, mostly through AI tools, with the intent of removing potentially harmful content more effectively and quickly (see, for instance, Gorwa et al,. 2020; Lim et al. 2020). These algorithmic moderation systems have been mostly analysed and assessed with reference to mainstream social media platforms, fuelled by growing public expectations for increased platforms’ responsibility. Overall, these systems are often criticised as being opaque and scarcely effective in complex sociotechnical contexts (Gorwa et al. 2020), as it can be very difficult for automated tools to make contextual decisions on complex and multifactorial concepts (Li and Williams 2018). Also, manual moderation does come with challenges. For instance, moderation is often carried out by freelancers in poor working conditions and exposed to extreme amounts of toxic content (Gillespie 2018).^3^ As noted, these considerations stemming from moderation research mostly come from analyses of mainstream platforms. Therefore, a research gap has been identified in considering the realities and needs of ad hoc, more specialised, platforms, such as those focusing on providing services for mental health and wellbeing.

In digital platforms focusing on mental health and wellbeing, moderation is often sided by different forms of counselling (e.g. moderators prompting at-risk users to access counselling services, or counsellors being active in online moderation). Digital counselling, despite its increasing popularity, is a service still considered complex and controversial (Hendry et al. 2017; Saha et al. 2020; Stoll et al. 2020; Perry et al. 2021; Barker and Barker 2022; Khan et al. 2022). In digitally mediate service encounters, counsellors deal with relatively new challenges, mostly linked to the increased accessibility and participation to the services offered, but also linked to the type of interactions they come across (which entail, for instance, reduced emotional proximity and the absence of non-verbal cues, see Bambling et al. 2008), their broader administrative tasks (Tummers et al. 2015; Breit et al. 2021), and risks of vicarious traumatisation (Furlonger and Taylor 2013).

Moderators and digital counsellors need both intellectual and social capital, as they require both specialised subject knowledge and the ability to navigate online support in effective ways (Mowlabocus et al. 2015, as discussed also in McCosker 2018). In this context, intellectual capital (subject knowledge) mainly refers to expertise in mental health. As a multidimensional concept, social capital can be defined as the connections amongst individuals and social networks, and the norms of reciprocity and trustworthiness that arise from them (see Putnam 2000:19). Being the glue holding together social collectives (Sum et al. 2008), social capital can facilitate the resolution of cooperative action problems (Coleman 1988; Putnam et al. 1994). At the core of this concept, is the idea that there are abilities and values rooted in social networks and relationships, and that these can be achieved through investment in social relationships; unlike the other forms of capital, no individual ‘owns’ these abilities and values, as they are only created through interactions across social networks (as summarised in Sum et al. 2008).

While recognising the pivotal role of moderators and digital counsellors, in framing mental health and recovery practices, it is important to recognise how their ability to act in certain ways is, in turn, framed by social media affordances, as they create possibilities that both enable and constrain action (Gibson 1977; Hutchby 2001; Bloomfield et al. 2010). Indeed, digital platforms, including those focusing on providing services for mental health and wellbeing, are best understood as sociotechnical assemblages and complex institutions (Gillespie, 2017), and can be conceptualised as composite human (users and, depending on the platform, moderators) and algorithm-driven non-human (automated tools and filters) entities embedded in their users’ general communicative practices (in line with Prochazka, 2019). As such, to fully understand the role of moderation and digital counselling, but also their challenges and possibilities, we cannot avoid considering the specific features of the platforms used (for instance, whether the communication is asynchronous or synchronous, whether it is organised according to threaded topics or time-based sequences, or the level of anonymity possible), as these aspects can directly affect communicative patterns and influence community cohesion, with implications in terms of the self-disclosure of members and their exchanges of social support (as discussed in Li et al. 2021). For instance, it has been suggested that, in synchronous communications (e.g. live chats), members of the community can communicate faster and, thus, form tighter connections; also, timely feedback seems to play a core role in fostering attachment between members, probably as speed works as a cue signalling support (Li et al. 2021). As such, we cannot ignore the importance of social media affordances in the context of social capital and, specifically, commitment, as digital spaces both enable and constrain certain behaviours, interactions, and even forms of thought (Ley 2007).

In what follows, we present our study, which furthers research on moderation and digital counselling to support users exhibiting risk behaviours by looking at the specific context of a specialised service, offering digital counselling and emotional wellbeing support to young people in the UK. In doing so, we explored the practices and perceptions of key actors (moderators, counsellors and individuals in key managerial roles), particularly in relation to the main challenges moderators face when performing their roles, with a specific attention to the identification of potentially risk behaviours, in the conversations where they provide support.

## Methodology

We interviewed a total of 6 Kooth.com’s Emotional Wellbeing Practitioners (tiered across trainees to more experienced individuals doing moderation and other agreed emotional wellbeing support with users), 3 Counsellors (who have a clinical and therapy accreditation by a professional body, and also perform moderation especially in high-risk situations), and 3 Subject Matter Experts (with responsibilities towards the community and its moderation or focusing on research and operations). Respondents have been identified in the article as PTSs 1–12, see Table 1.

**Table 1:** Interviewees

Interviewee	Role at Kooth
PTS1	Emotional wellbeing practitioner
PTS2	Emotional wellbeing practitioner
PTS3	Subject matter experts
PTS4	Emotional wellbeing practitioner
PTS5	Emotional wellbeing practitioner
PTS6	Counsellor
PTS7	Emotional wellbeing practitioner
PTS8	Counsellor
PTS9	Emotional wellbeing practitioner
PTS10	Subject matter experts
PTS11	Counsellor
PTS12	Subject matter experts

Interviews took place through 4 individual interviews (with PTS6, PTS7, PTS8 and PTS9, respectively) and 3 focus groups (firstly, with PTS1, PTS2 and PTS3, secondly with PTS4 and PTS5, and lastly with PTS10, PTS11 and PTS12) carried out in October and November 2021, and in March 2022. Convenience sampling was used during the recruitment. Accordingly, interviews and focus groups were scheduled to suit participants’ availability. Since not all Kooth professionals could take part in the research on the same date and at the same time, both interviews and focus groups were conducted. Furthermore, to ensure that the data collection process was as efficient, smooth and convenient as possible, participants were recruited with the support of the organisation's Research and Evaluation Lead, who advertised the opportunity to engage voluntarily. Moderators were facilitated to attend during their work.

The in-depth interviews were carried out online through the platform Teams, and video-recorded to keep track also of non-verbal cues. The audio (for a total of 5.30 h) was then transcribed and anonymised, in line with the procedure approved by the Ethics Committee of the University of Southampton (ethical approval ref ERGO/FEPS/66387). The interviews and focus group discussions were semi-structured, thus followed a pre-defined guide, on the basis of the project’s research questions. Slides with key queries were shared with the participants to facilitate their flow, and to remind participants what they were asked.^4^

All the transcribed material was manually coded (directed content analysis—see Hsieh and Shannon 2005) according to the coding scheme summarised in Table 2 (with codes identified a priori*,* in light of our research aim, and subcodes partially adjusted throughout the analysis), and then organised in the following main themes: roles and responsibilities; risks; what works; and current challenges.

**Table 2:** Coding framework

Codes	Subcodes
The actor	Self-definitions; previous/parallel experience; Background; tasks; challenges; training received; support received
The work	Specialisation; shifts; team; what works; what can be improved and how; numeric indications (users to deal with/shift; submissions/quarter, etc.); Stages of the work; rating system
The platform	What works; what can be improved; potential issues with (semi)automatization
Risk behaviours	Physical health; self-harm; sexual health; mental health; other
Other	Emerging issues/proxy indicators of problems; COVID-19; links to criminal behaviour/gangs

Two researchers (Author 1 and 2) undertook the coding ensuring inter-coder consistency (Sanders and Cuneo 2010; O’Connor and Joffe 2020), while the project PI (Author 3) was involved in the writing-up of the results and contributed to the refinement of this contribution. Any difference in interpretation among the researchers was addressed through discussions, and clarifications—if needed—were sought by engaging with the Research and Evaluation Lead at Kooth Plc. For practicality, the coding procedure was conducted manually, using a Word document on which every relevant portion of transcription was highlighted, according to a previously agreed colour-coding scheme. Whilst colours were used to indicate codes, comments were employed to signal subcodes. This unorthodox strategy was chosen after a discussion with other members of the multidisciplinary research team (to be involved in other stages of the project) and Kooth, as it allowed researchers from different backgrounds, at times non-familiar with qualitative research and coding strategies, to access and monitor the annotated dataset without having to access specialized software.

While extensive reflections on the benefits and the challenges of working in multidisciplinary research teams, and in having representatives of the organization object of the analysis as part of the broader research team in the underlying project (*SafeSpacesNLP*—see in the Introduction) would exceed the scope of this contribution, it is important to briefly underline how these aspects had a direct impact on our research design. First of all, it is important to note that, in order to avoid biases, as regards the study presented in this contribution, Kooth’s representatives were involved only as gatekeeper to facilitate access to potential respondents, and in helping the researchers to clarify some aspects regarding organizational aspects at Kooth; no direct input was given on the data collection or analysis process. However, because of Kooth involvement in other stages of the project, the Research and Evaluation Lead at Kooth was able to provide constructive feedback on the research design of the broader project, and he was involved in the ethical oversight of the project and compliance with our data sharing policy throughout.

## Results

### Roles and responsibilities

From the interviews, the complexity, sensitivity and fluidity of the roles of Emotional Wellbeing Practitioner, Counsellor and Subject Matter Expert clearly emerged. First of all, their tasks are performed in a multi-platform and multi-layered environment (referred to as ‘the platform’ in the article), with relevant information coming from (synchronous and asynchronous) chats, direct messages, a users’ forum comprising a discussion board and articles (whose posts and comments are moderated), users’ personal journals and written goals, and a service inbox. Additionally, there is a dedicated instant messaging channel for staff to exchange information and get help and clinical support from colleagues and senior shift leads, in what has been described consistently as a ‘*nurturing environment*’ (PTS2). In order to keep track of everyone’s work, a dedicated spreadsheet is used (‘*so that we're not stepping on each other’s toes and not all clustering is in one area*’, PTS2) to record the team and service tasks. In this way, moderators and counsellors can focus on certain textual information or on specific users (some users, for instance, have a named worker allocated to them), in line with their seniority, when there are at-risk situations.

Some tasks have been especially difficult to quantify for interviewees, as they can change every day. These included the number of submissions they had to address, which depending on level of risk and complexity could vary from 10 to 100 per shift. Similarly, while caseloads are set for 1:1 chats, other types of moderation can be more fluid, for instance when moderating live a discussion board (a type of service-led discussion forum scheduled at a specific time). The fluidity of the work depends on the specific features of the digital services available which can offer a combination of synchronous and asynchronous communication. For example, while users can submit their journals or send comments in forum discussions 24/7, the synchronous chat with practitioners is open at specific times only. Depending on the role and the seniority, alongside counselling and moderation, time is allocated to specialised training or to administrative and managerial tasks.

Interactions with users are strictly regulated clinical governance processes, both as regards access to certain services (particularly live chats with Counsellors and Emotional Wellbeing Practitioners), and as regards content. For instance, there is a shared resource document with ‘*what we're allowed to send to young people, so it's a big list of, like, websites and NHS resources, different organisations, that we can kind of send links to*’ (PTS7); specific goals (e.g. contacting their GP) are to be set or reviewed in each chat; or end-of-chat messages are to be sent as an encouragement, a recap of the session, or reminder of any goals set or helpful strategies discussed.

A significant part of the moderators’ task concerns enabling forms of peer support (‘*A lot of the time they'll talk about things that aren't risky though, and that's about maintaining their wellbeing another way. So they might talk about their favourite TV show or something, and in that case we are probably not interacting with them, just kind of publishing that post and facilitating them getting that peer support*’ (PTS3)) and making sure that the content shared with other users is appropriate (for instance, moderators might have to edit posts and comments keeping them ‘*as close to how the young persons express themselves as possible but de-escalating risk, […] maybe deleting some bits and then publishing it*’ while interacting with the user privately (PTS1)). Content moderation is guided by users' age: there are different age ratings and, as explained by the respondents, *‘there shouldn't be any interaction between those age ranges to keep people safe obviously, and the kind of things that people are discussing, the life experience is very different’* (PTS9); ‘*what may be suitable for [some] might not suitable for [younger people], so we've got to double check*’ (PTS1).

Depending on the level of risk evidenced in the conversations, ‘*the engagement levels change*’ (PTS1) users might be referred to a specialist (‘*As moderators we don't tend to do the deeper, the therapeutic side of things […] We tend to focus more on trying to get them into the team, you know, like to the counsellors’* (PTS6)). Counselling takes place digitally and is text-based (even if, in some parts of the country, there is the possibility to access face-to-face counselling).

The workforce employed is pluralistic, with expertise in mental health for young people, coming both from specialised educational backgrounds (many are qualified counsellors, or counsellors in training) and from diverse types of mental health practical experience (e.g. ‘*I have actually been moderating mental health communities online for about 20 years outside of Kooth*’ (PTS1); ‘*I've done a lot of work in schools […] and as a support worker, kind of building up to doing my counselling qualification*’ (PTS8)).

The complexity and fluidity of the context, not surprisingly, creates a series of role tensions, with Emotional Wellbeing Practitioners, Counsellors and Subject Matter Experts alike having to juggle different needs. A main aspect refers to their own wellbeing (as tasks can be ‘*emotionally draining*’ (PTS4)):

‘It's not the it's not just dealing with the one risk, it's dealing with multiple bits of risk and it all just kind of layering on and then by the end of the day you just like ‘Jesus, that was a lot of heavy different topics on a load of different stuff as well’ [...] But then there's some that bring you right back and they hit you right in your stomach and you really feel them and they can impact you afterwards’ (PTS7).

All respondents discussed the importance of self-care and of setting their own boundaries, both by themselves (‘*I like lighting a candle at eight o'clock. So, lighting a candle, I'm putting my music on. And that's how I… get through my shift*’ (PTS5)), or through peer support (‘*The peer support we provide each other as colleagues and […] having awareness of things like burnout and vicarious trauma […] I can probably see the word suicide 100 times a day if I'm not careful, that kind of thing. So kind of prolonged and chronic and that kind of exposure*’ (PTS3)). Additionally, those involved in moderation have regular meetings with their line managers and can access clinical support and external supervision.

A second important aspect refers to the need to manage time effectively through complex situations, especially after performance indicators (used for benchmarking) were introduced in recent years. That change, in the words of some respondents, ‘*added pressure*’ (PTS5), creating some ‘*rush*’ that can be difficult to manage when ‘*dealing with emotional […] wellbeing and trauma’* (PTS4).

### Risks

Not surprisingly, assessing risk is central in moderators and counsellors’ activities (‘*So it's almost a constant, every minute that we're working we're assessing for risk in one way or another from multiple directions*’ (PTS1)), in what has been described as a ‘*better safe than sorry*’ approach (PTS2). During every shift, a couple of moderators oversaw scanning messages for risks, to escalate those needing more urgent attention in the platform.

As collectively reported by the respondents, risks in Kooth.com often relate to eating disorders, anxiety, depression and other types of mental health issues, gender dysphoria, sexual health, self-harm behaviours, suicide attempts, bullying, physical and mental abuse, sexual exploitation, forced marriages, and grooming. Also, victims of crime or young people exploited in crime (for instance, young people involved in gangs for drug dealing) report their experiences online. These risks can also be multiple, and some of those risks worsened during COVID-19 restrictions (‘*we’ve just seen numbers go through the roof*’ (PTS3)—see Gerrard 2020, on the surge in demand for mental health charities during the pandemic). However, as reported by one respondent, in many cases the risks identified are ‘*the early levels, so kind of early emerging eating difficulties [or] we're seeing situations escalate, so maybe it's a situation with bullying that is becoming physical*’ (PTS3).

There can be cases where a risk has already escalated, or there is an imminent danger (and, as such, external services are called: ‘*So if they disclose that they've self-harmed badly, taken an overdose or a severe risk, we can call ambulances for them*’ (PTS3)). And some users could be more at-risk than others. The service has multiple processes in place to quickly communicate to staff what level of risk someone is currently assessed at, and any key aspects of their care plan to be aware of.

### What works

Overall, the respondents were very positive regarding the social utility of their work, as evidenced for instance in the following snippet: ‘*Even if it takes us a bit of time, we're still far faster than unfortunately people like GPs or CAMHS*^5^* can be at the moment, so we are still providing, you know, waiting list free, essentially, access to not just support, but to be able to flag up issues and then have them kind of escalated and dealt with professionally*’ (PTS3). Even in less at-risk cases, they can give users ‘*that little bit of the extra confidence, just to kind of make that step for themselves because they've seen other people’s personal experiences of that*’ (PTS7).

The approach used is considered effective, as it allows a good mixture of both peer and professional support (‘*We've seen the amazing support that these kids give each other on that website, it's just absolutely fantastic*’ (PTS2); ‘*We keep that a nice community and nice place for them all to speak*’(PTS6)). And, reportedly, the feedback received from users is very positive as well.

Personal experiences in the organisation were generally valued positively, especially as regards team support and the possibility to have some build-in flexibility in their tasks (e.g. ‘*We are an agile little team […] It is a very robust system, people are very, very supportive and we work in a really collaborative way*’ (PTS3).

### Current challenges

In the previous section, titled ‘what works’, we pointed out approaches and practices that responsible AI systems should maintain, foster and further implement. Building on that discussion, in this section we discuss a number of existing challenges and difficulties identified by our respondents, which are of particular interest in the context of this study as they highlight necessary points of intervention. The challenges reported by the respondents were grouped into the following topics: time use and multitasking; reading in between the lines; and more subtle risks.

#### Time use and multitasking

Time is a scarce resource, and—because of the volume of work—respondents consistently mentioned (the lack of) time as a major issue in carrying out their tasks at their best, leading to the lack of taking sufficient breaks or allocated time to debrief (*‘it was just too much because I couldn't really process in between chats*’ (PTS8)), especially when there are risk situations (for instance, suicidal intention) (‘*a risky situation can take up a whole shift, especially if there is a immediate risk for the user’s safety*’ (PTS4)). Moreover, time issues can lead to less effective support, for instance when users are left waiting too long to access digital counselling, or when errors can be made because of necessary multitasking and difficulty of the task (*‘With a high volume of work, there are going to be errors […] You know, somebody misses, like I say, like a case note, or they might not have edited…’* (PTS9); ‘*I've moderated a post that […] it was not against boundaries, but it did kind of push the boundaries a little bit. […] I was dealing with multiple things at once when I read it I was like ‘that's that's within the boundaries, I'll publish that’, like the wording wasn’t off, but because it was so short, like you missed their underlying tone, 'cause sometimes it's not necessarily what's written there, but it's kind of the impact that would have on the community as a whole, it's like an ecosystem at the end of the day we want a positive one and so’* (PTS7)). Of course, clinical auditing processes are regularly carried out to understand, identify and mitigate human error, including processes that take into consideration the platform and the ecosystem of services that are delivered.

As explicitly discussed by some respondents, a system to help them navigating the mole of information they need to go through would be welcomed, as exemplified in the following snippets:

‘At the moment a lot of our processes are very manual and us literally just sitting there and reading through kind of really large chunks of text, so I don't know something that made that those couple of words [e.g. suicide] pop out a bit so they don't get missed and are easier to pick up’(PTS3).

‘Time [...] is a big challenge [...] 'cause sometimes you're trying to, you know, keep all the immediate posts, you know, spend your time on them, but then also you've got [...] the lower risk posts which are just as important because they want to be heard, they need the peer support [...] They sometimes might be [temporarily] left because risk always comes first [...]. So I don't know if there could be a tool to identify, you know, high risk posts’ (PTS6).

#### Reading in between the lines

Identifying risk is not always straightforward, as users might be less ‘direct’ in expressing their feelings and emotions, and there are no visual or non-verbal cues to be observed (there are, however, text-based cues). For instance, users could use metaphors (PTS4), or post poetry that needs to be interpreted (PTS2). As such, both moderators and counsellors need to try and slowly build the picture of what is going on, especially since users can sip the amount of information they provide (*‘they[users] are definitely in charge*’ (PTS4)), and, online, many important risk-relevant cues used in other contexts (such as the body language, the tone of the voice) are missing (‘*When I first started doing chats, I used to have a massive headache all the time […] Swapping over from doing face to face interventions, or even telephone counselling or telephone counselling skills is like massive*’ (PTS8)). Consider the following example:

‘A young person could say I have been feeling very low lately… I’ve got a lot going on in my life, I'm feeling scared, I’m feeling alone. [...] Straight away as moderator I would think: well, why are they feeling scared, why do they feel alone, what's going on in their life?. That could be anything, [...] there could be risk there [...] As moderators we go digging, we want to find out a lot more [...] You can open a can of worms unknowingly’ (PTS6).

#### More subtle risks

Finally, there are some moderation and counselling challenges that are linked to what could be considered more subtle forms of risk (to the individual user, or to the digital community), and that can complicate the work of employed staff, or hinder the inclusiveness of the service provided. Some have to do with organisational issues (because of the resources available): for instance, despite the extensive operational hours of the services offered at Kooth,^6^ it was lamented by one of our respondents that Emotional Wellbeing Practitioners and Counsellors are not available overnight and are available only at limited times over the weekends, which might discourage some potential users from participating, as they might have less time during the rest of the week (PTS8).

More challenges are linked to the content posted, as extra attention is constantly needed to make sure not only that shared content is appropriate, but also appropriate to a specific age group (‘*It's acceptable for a 16 year old on our site to ask where they can get free condoms, but if an 11 year old is asking that question we are reacting in a very different way*’ (PTS3)). Additionally, attention is constantly needed to ensure that the digital platforms do not foster any type of dis- or mis-information (‘*We are very aware we're not medical professionals and their peers certainly aren't either so with those questions what we tend to do is we'll say: we're really sorry we can't publish this […] but hey let's have a talk about it, maybe here's an NHS information page we can give you that you can read and come back if you have any questions’* (PTS3)). Also, anonymity and confidentiality must be maintained throughout, so that extra effort is needed to check, for instance, that the same username is not used across social media as that could easily reveal the real identity of a user (‘*Something that could be useful to do with some automation if possible*’, PTS1). At times, the content posted could be difficult to interpret because slang (from different geographical areas) is used, or users make references to numbers (for instance, of county lines offences) and moderators could not be familiar with those (PTS2).

A final set of risks refers to the need and importance to foster a climate of trust with users, and to build and maintain a relationship (‘*you need to build that rapport with them first, before they feel that they can open up*’ (PTS5)). This entails showing that staff care about users, but also that they need to be treated with respect (‘*You know, we sometimes get asked “are you robots, like are you real people?”*’ (PTS3); ‘*You do get some users who sign up and they just send like erm, ridiculous things. And, or they think that we're like robots. […] Yeah, or like those and rude things, or……very inappropriate things. [..] We always message them in a way that shows that we are human, […] a lot of the time, the users who send something silly, they're just kind of testing the water*’ (PTS9)).

## Discussion and conclusions

Digital platforms—as intermediaries bringing together users, service providers, content producers and other stakeholders for a range of social exchanges (Srnicek 2017)—are of increasing social importance, to the point that it has been claimed that we now live in a ‘platform society’ (van Dijck et al. 2018). This society, however, is generally dominated by large scale, monopolistic platforms, and so is most research on digital moderation. In the study presented here, we focused on the contrary on the realities and needs of a specialised platform focusing on providing services for mental health and wellbeing to young people.

As discussed previously, the main goal of the study reported in this article was to further research on moderation and digital counselling to support users exhibiting risk behaviours by looking at the practices and perceptions of key actors involved in moderation, counselling, or in managerial roles. We were particularly interested in the main challenges those involved in substantial moderation practices (in our case, Emotional Wellbeing Practitioners and Counsellors) face when performing their roles, to create a benchmark to ideate and develop ways to improve the system currently in place, exploring ways in which responsible AI solutions can support and better target interventions for young people asking for help or engaging in risk behaviours online.

We have seen that Kooth effectively combines individual counselling with community support and is subject to rigorous ethical standards. If we are to address the thorny issue of safe moderation within digital platforms, it is vital that we learn from well-established services in the field to specifically understand the key challenges and barriers to safe, scalable moderation. To summarise, the challenges identified by counsellors and moderators we interviewed mainly revolved around three areas: time management, interpretation of user communication, and subtle risks. The first category of challenges involved the time constraints imposed by the limited resources available, despite the number of young people turning to the online platform for support. Such time challenges were exacerbated by the type of support provided and the difficulties involved by reducing the time dedicated to the discussion of sensitive topics with vulnerable users needing assistance. At the same time, these challenges also caused professionals to limit their time to filter the information provided by the users, and to recover after these difficult discussions. The second group of challenges regarded how users communicate on the platform and with the professionals available on it. Since struggles and needs are often communicated indirectly, calls for support and their urgency are not immediately clear from the user interactions and frequently require mental health workers to interpret the texts received through the platform and to collect more information about individuals who authored them, to better understand their communication styles and preferences. Given the sensitivity of the topics and risks, these tasks require more time and effort from the moderators and counsellors to process relevant data and accordingly respond to users. The last set of challenges mentioned during the interviews dealt with the need professionals have to build and maintain a trustworthy relationship with the young people they support to be able to help them to the best of their abilities. At the same time, workers also needed to ensure the suitability of all contents that are publicly shared through the platform, according to viewers age, vulnerability, and cognitive capacities. Therefore, all the challenges discussed by counsellors and moderators are intertwined and interrelated.

Recent advances in responsible AI methods are now providing insights into possible solutions to some of these challenges. With regards the challenge of time use and multitasking, modern text classification algorithms (Minaee et al. 2021) can classify large volumes of online posts to allow moderators to better triage and filter posts, and to flag time critical posts for example those that could represent a threat to life such as posts with markers associated with suicidal behaviour or immediate need. The use of AI to organise content can also provide useful structure to moderator's daily workload, reducing the chance of human error due to missed or forgotten content. The use of AI to classify ambiguous, subtle or contextual age-appropriate content has led to various grand challenges within the AI research community (Joshi et al. 2017), but AI approaches that can embed knowledge graphs containing common sense and/or domain-specific contextual knowledge into AI models may yield results in the medium term. Recent research projects are exploring a range of novel AI methods to progress text classification research. For example, SafeSpacesNLP^7^ and ProTechThem^8^ are exploring AI models that can move beyond single post classification, such as hate speech classification, and towards an ability to identify moments of change within conversations around mental health issues. Other projects such as the UKRI TAS Trust Node^9^ and EU H2020 project WeVerify^10^ are exploring AI models for trustworthiness and misinformation detection. A recent trend is the emergence of new human-in-the-loop AI approaches (Middleton et al. 2022), which represents an exciting direction of travel as ultimately online content moderation is a human process and we need AI algorithms which can be trustworthy and supportive to empower human decision makers to concentrate more time on the subtle and tricky moderation decisions which AI algorithms find hard to process, leading to an use of automation that can enhance human activities in moderation to preserve a safe space and positive digital mental health community.

As such, as responsible AI approaches are becoming increasingly promising in their capacity to support up-scaling of existing manual moderation approaches and better target interventions for vulnerable users in sensitive settings, whilst developing in ways that are ‘fit for purpose’ and minimise potential biases. Sociotechnical approaches bringing together computational expertise with social sciences and subject matter experts' ability to investigate and interpret qualitative datasets is becoming essential. Looking forward, in our opinion it is the combination of human experience, with its capacity for insight, connection and empathy, alongside responsible AI, with its capacity for processing content at scale, that will deliver the step changes needed to address the significant challenges we have identified for digitally scaled-up moderation in a digital mental health community. If AI is delivered in a responsible way, with safeguards in place as part of a wider and evidenced governance framework, coupled with opportunities for stakeholder trust to be built via mechanisms such as codesign, then it can potentially empower moderators and lead the field of digital community moderation into an exciting future.

## Footnotes

1 https://www.tas.ac.uk/safespacesnlp/.

2 https://www.koothplc.com/.

3 It is noted that this is not the case for the organisation targeted in this study.

4 First, each participant was asked to introduce themselves. Specifically, they had to comment about their role within Kooth, average workload and challenges frequently faced in their jobs, and the nature of their roles. This first part of the plan was meant to provide a background to the professional role of the participants. Then, a series of questions focussed on risk behaviours they had to identify, moderation practices and forms of interventions, temporal sequencing of actions, professionals involved, reporting or record-keeping, temporary urgency and strategies typically used to respond to it, as well as indicators Kooth councillors and moderators tended to look for in young users they supported online. Finally, specific examples were asked, which served to provide supporting evidence and clarity to the points made by the participants.

5 General Practitioners (GP), in the UK are doctors who treat all common medical conditions and refer patients to hospitals and other medical services; Children and Adolescent Mental Health Service (CAMHs) in the UK are the public services that assess and treat young people with emotional, behavioural or mental health difficulties.

6 Kooth operates out of hours service weekdays from 12 to 10 pm and the weekend from 6-10 pm 365 days a year.

7 https://www.tas.ac.uk/safespacesnlp/

8 http://www.protechthem.org/

9 https://trust.tas.ac.uk/

10 https://weverify.eu/
